# A New Archosauriform (Reptilia: Diapsida) from the Manda Beds (Middle Triassic) of Southwestern Tanzania

**DOI:** 10.1371/journal.pone.0072753

**Published:** 2013-09-27

**Authors:** Sterling J. Nesbitt, Richard J. Butler, David J. Gower

**Affiliations:** 1 Burke Museum and Department of Biology, University of Washington, Seattle, Washington, United States of America; 2 GeoBio-Center, Ludwig-Maximilians-Universität München, Munich, Germany; 3 Department of Life Sciences, The Natural History Museum, Cromwell Road, London, United Kingdom; State Natural History Museum, Germany

## Abstract

**Background:**

Archosauria and their closest relatives, the non-archosaurian archosauriforms, diversified in the Early and Middle Triassic, soon after the end-Permian extinction. This diversification is poorly documented in most Lower and Middle Triassic rock sequences because fossils of early groups of archosauriforms are relatively rare compared to those of other amniotes. The early Middle Triassic (? late Anisian) Manda beds of southwestern Tanzania form an exception, with early archosaur skeletons being relatively common and preserved as articulated or associated specimens. The Manda archosaur assemblage is exceptionally diverse for the Middle Triassic. However, to date, no non-archosaurian archosauriforms have been reported from these rocks.

**Methodology/Principal Findings:**

Here, we name a new taxon, *Asperoris mnyama* gen. et sp. nov., from the Manda beds and thoroughly describe the only known specimen. The specimen consists of a well-preserved partial skull including tooth-bearing elements (premaxilla, maxilla), the nasal, partial skull roof, and several incomplete elements. All skull elements are covered in an autapomorphic highly rugose sculpturing. A unique combination of character states indicates that *A. mnyama* lies just outside Archosauria as a stem archosaur within Archosauriformes, but more precise relationships of *A. mnyama* relative to other early archosauriform clades (e.g., Erythrosuchidae) cannot be determined currently.

**Conclusions/Significance:**

*Asperoris mnyama* is the first confirmed non-archosaurian archosauriform from the Manda beds and increases the morphological and taxonomic diversity of early archosauriforms known from the Middle Triassic. The direct association of *A. mnyama* with species referable to Archosauria demonstrates that non-archosaurian archosauriforms were present during the rise and early diversification of Archosauria. Non-archosaurian archosauriforms and archosaurs co-occur in fossil reptile assemblages across Pangaea from the late Early Triassic to the end of the Late Triassic.

## Introduction

Archosauria, the crown clade that includes living birds and crocodilians as well as extinct dinosaurs, pterosaurs and pseudosuchians (stem-crocodilians), is one of the most successful evolutionary radiations in the history of vertebrate life on land [1]–[3]. Archosauria is part of a wider evolutionary radiation, Archosauromorpha, that includes also extinct taxa more closely related to them than to lepidosauromorphs (lizards, snakes, rhynchocephalians and closely related extinct taxa) [4]. Within Archosauromorpha, Archosauria and its closest extinct relatives form the clade Archosauriformes ([5],  =  Archosauria of Benton in [6]), all of which possess anatomical features that were classically considered as key archosaur characters (e.g. presence of the antorbital fenestra in the skull). Whereas the origins of archosauromorphs extend into the latest Palaeozoic (e.g. [7]), the first non-archosaurian archosauriforms appear around the Permian–Triassic boundary (c. 252.6 Ma; [8]), and are more commonly recovered from Early and early Middle Triassic rock sequences (e.g. [9]–[13]). The first archosaur body fossils appear in the fossil record around the boundary between the Lower and Middle Triassic (c. 247 Ma; [14], [15]), and the group subsequently radiated dramatically, becoming the ecologically dominant large-bodied vertebrate group in terrestrial ecosystems by the end of the Triassic [8], [16], [17]. By contrast, non-archosaurian archosauriforms seemingly display reduced phylogenetic diversity but increased ecomorphological specialization (e.g., *Vancleavea campi*, proterochampsids) in terrestrial ecosystems from the late Middle Triassic onwards, and did not survive the end-Triassic extinction event.

The late Early and Middle Triassic thus mark a period of major transition from assemblages dominated in abundance by non-archosaurian archosauriforms to assemblages dominated by archosaurs. In some parts of the world, this transition is marked by co-occurrences of non-archosaurian archosauriforms and archosaurs at a single site, or within a single rock package ([12]; see discussion).

The most important currently known fossil assemblage of early archosaurs comes from the Lifua Member of the Manda beds of southwestern Tanzania. Fieldwork conducted by British, German and American-led teams in the Manda beds since the 1930s has yielded a diverse series of early Middle Triassic (Anisian) vertebrates [18]–[20]. This assemblage includes at least seven early archosaur species, several of which remain unnamed, including the earliest known dinosauromorph [20], a possible dinosaur [21], and a minimum of five pseudosuchians [22]–[30]. However, to date no non-archosaurian archosauriform has been described from the Manda beds.

Here we describe the first non-archosaurian archosauriform from the Manda beds, and erect a new genus and species for it on the basis of its unusual cranial morphology. This new taxon provides novel insights into the morphological and phylogenetic diversity of early archosauriforms and the Manda assemblage during the transition from early archosauriforms to early archosaurs.

### Specimen history

**Figure 1:**
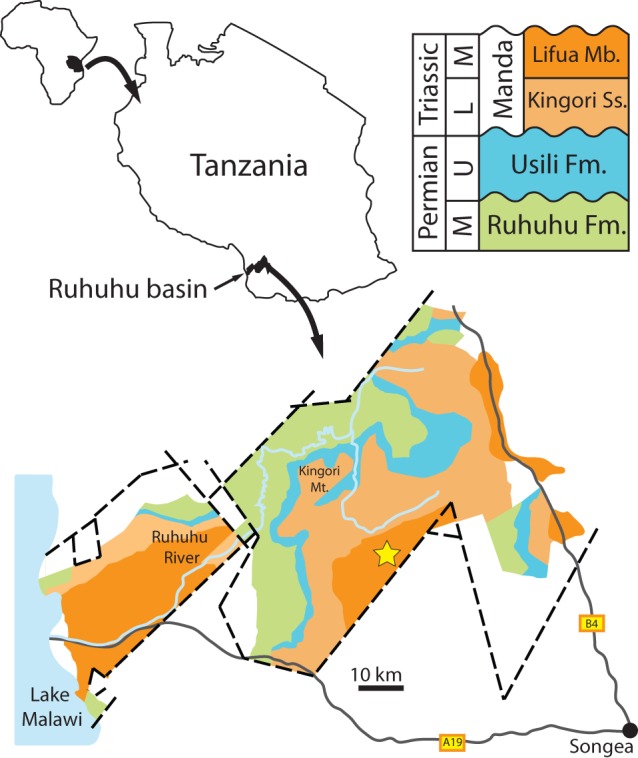
Holotype locality of *Asperoris mnyama* (NHMUK PV R36615) in the Lifua Member of the Manda beds, Ruhuhu Basin, southwestern Tanzania, Africa. Star indicates the approximate position of the holotype locality of *Asperoris mnyama*. Outcrop area modified from Stockley (1932). **Abbreviations**: Fm, formation; L, lower; M, middle; Mb, member; Mt., mountain; Ss., sandstone; U, upper.

The only known specimen of *Asperoris mnyama* was discovered during the 1963 British Museum (Natural History) (now the Natural History Museum, London) – University of London Joint Palaeontological Expedition to northern Rhodesia (now the eastern portion of Zambia) and Tanganyika (now the western portion of Tanzania) (see [18]). The holotype was found and collected on August 23^rd^, 1963, (Cox unpublished fieldnotes, NHMUK) in the Lifua Member of the Manda beds in the Ruhuhu Basin of southwestern Tanzania (Fig. 1). The specimen, designated U9/1 under the locality recording system of the expedition, was collected between the town of Litumba Ndyosi and the mountains to the immediate west at locality U9. This locality was described as being “south of the Njalila river near the Njalila-Litumba road” (Cox unpublished fieldnotes, NHMUK), and fragmentary postcranial remains of dicynodonts (uncatalogued at NHMUK; SJN pers obs) and other amniote remains were found in the local area (uncatalogued at NHMUK; SJN pers obs). U9/1 was mapped more precisely by Cox [31] as locality “9” lying between the Njalila and Hiasi rivers (Fig. 1 of [31]) in the drainage of the Hita River (derived from local knowledge, 2007, SJN). Unfortunately, no detailed geological information was recorded for U9 but other localities in the immediate area are characterized by fluvial sediments consisting of sandstones and red mudstones [19].

No specimen preparation notes have been found, but we deduced the following from close examination of the material and additional preparation. As evidenced by slight residues in tiny holes and the uneven dissolution of calcitic veins along the surface, the specimen was prepared in an acid bath (acid type, concentration, and immersion time unknown) to reveal fine details of the bone surface. The bone surface is exquisitely preserved and the matrix easily separated in most cases. After the acid bath, some of the freed elements (e.g., premaxilla and maxilla) were reassembled and rearticulated by the addition of an unidentified compound composed of ground paper and an acetone-soluble bonding agent. This bonding agent has now been removed by preparation staff at the NHMUK as part of our re-examination of the specimen. One of us (SJN) conducted additional preparation in order to define morphological features by removing a medium course sandstone composed of rounded quartz cemented by calcium carbonate using an ARO Marxall pneumatic airscribe modified with an HW-10 (HW [Hardy Winkler] Co.) microtip adaptor. Butvar B-76 dissolved in acetone was applied with a small brush to protect the surface following detailed surface preparation, and fragments previously taken apart were reassembled using Paraloid B-72 dissolved in ethanol. To enhance photographic detail, each fragment of the specimen was covered in a thin coat of water-based neutral gray paint (Winsor & Newton Designers Gouache, Neutral Gray #3) and immediately washed away after photography.

### Institutional abbreviations

BP, Evolutionary Studies Institute of the University of the Witswatersrand (formerly the Bernard Price Institute for Palaeontological Research), Johannesburg, South Africa; GPIT, Paläontologische Sammlung der Universität Tübingen, Tübingen, Germany; GR, Ruth Hall Museum of Paleontology, Ghost Ranch, New Mexico, USA; IVPP, Institute of Vertebrate Paleontology and Paleoanthropology, Beijing, China; NHMUK, The Natural History Museum, London, United Kingdom; NMT, National Museum of Tanzania, Dar es Salaam, Tanzania; PIN, Paleontological Institute of the Russian Academy of Sciences, Moscow, Russia; SAM, Iziko South African Museum, Cape Town, South Africa; SMNS, Staatliches Museum für Naturkunde, Stuttgart, Germany; TTU-P, Museum of Texas Tech University, Lubbock, TX, USA.

## Methods

No permits were required for the described study, which complied with all relevant regulations.

### Nomenclatorial acts

The electronic edition of this article conforms to the requirements of the amended International Code of Zoological Nomenclature, and hence the new names contained herein are available under that Code from the electronic edition of this article. This published work and the nomenclatural acts it contains have been registered in ZooBank, the online registration system for the ICZN. The ZooBank LSIDs (Life Science Identifiers) can be resolved and the associated information viewed through any standard web browser by appending the LSID to the prefix “http://zoobank.org/”. The LSID for this publication is: urn: lsid: zoobank.org: pub: E510913F-AE48-494B-80ED-2D6CC5AAF74A. The electronic edition of this work was published in a journal with an ISSN, and has been archived and is available from the following digital repositories: PubMed Central, LOCKSS.

### Phylogenetic analysis

We added the new taxon to the archosauriform phylogenetic dataset of Nesbitt [8] (see table 1). This dataset focuses primarily on Triassic archosaurs with some sampling of stem archosaurs (i.e. some non-archosaurian archosauriforms). Taxon sampling of non-archosaurian archosauriforms in this analysis is limited, excluding for example several possible members of Proterosuchidae and Erythrosuchidae. Addition of these taxa will be needed in future following ongoing systematic and anatomical revision of these taxa [10]. Nevertheless, Nesbitt's [8] dataset is considered sufficient to determine whether the new taxon belongs to crown-group Archosauria or is a close relative.

**Table 1:** Character scores for *Asperoris mnyama* added to the dataset of Nesbitt [8].

?0030	10000	000??	00000	?0000	010??	0?0?0	????0	00?0?	?????
?????	??00?	001?0	?????	?????	?????	?????	?????	?????	?????
?????	?????	?????	?????	?????	?????	?????	11?00	??001	00--?
?????	?????	?????	?????	???1?	?????	?????	?????	?????	?????
?????	?????	?????	?????	?????	?????	?????	?????	?????	?????
?????	?????	?????	?????	?????	?????	?????	?????	?????	?????
?????	?????	?????	?????	?????	?????	?????	?????	?????	?????
?????	?????	?????	?????	?????	?????	?????	?????	?????	?????
?????	?????	?????	?????	?????	?????	?????	?????	?????	?????

The new dataset comprised 79 terminal taxa and 412 characters, and analysis was similar to that of Nesbitt [8]. The new taxon was scored for 47 of the 412 characters. Eighteen characters (32, 52, 121, 137, 139, 156, 168, 188, 223, 247, 258, 269, 271, 291, 297, 328, 356, 399) were treated as ordered and the non-archosauriform archosauromorph *Mesosuchus browni* was set as the outgroup. We rescored character 4 for *Erythrosuchus africanus* from 1 (in Nesbitt 2011) to 3 based on a reexamination of well preserved material. A heuristic search was performed with 1000 random addition (RA) replicates using tree bisection and reconnection (TBR) branch swapping as implemented in PAUP* v4.0b10 [32]. Branches with a minimum length of zero were collapsed. The nexus file is available in the supplementary data.

## Results

### Systematic paleontology

Reptilia Laurenti, 1768 sensu [33].

Diapsida Osborn, 1903 sensu [34].

Archosauromorpha Huene, 1946 sensu [4].

Archosauriformes [5].

*Asperoris mnyama* gen. et sp. nov.

urn: urn: lsid: zoobank.org: act:621B690B-3D20-46F0-896B-7B5237A86E81

#### Etymology

*Asper*- (L. *asper*, “rough”), *oris* (L. oris, “face”). This combination refers to the aberrant sculpturing on the surface of the skull bones; *mnyama*, Swahili for beast.

#### Holotype

NHMUK PV R36615, well-preserved incomplete skull including much of the right maxilla, nearly complete right premaxilla, much of the right nasal, ventral process of the postorbital, right prefrontal, right frontal, right parietal, much of right postfrontal, other unidentified skull fragments (Fig. 2). This is the only known specimen of the species (Figs. 2–12).

**Figure 2:**
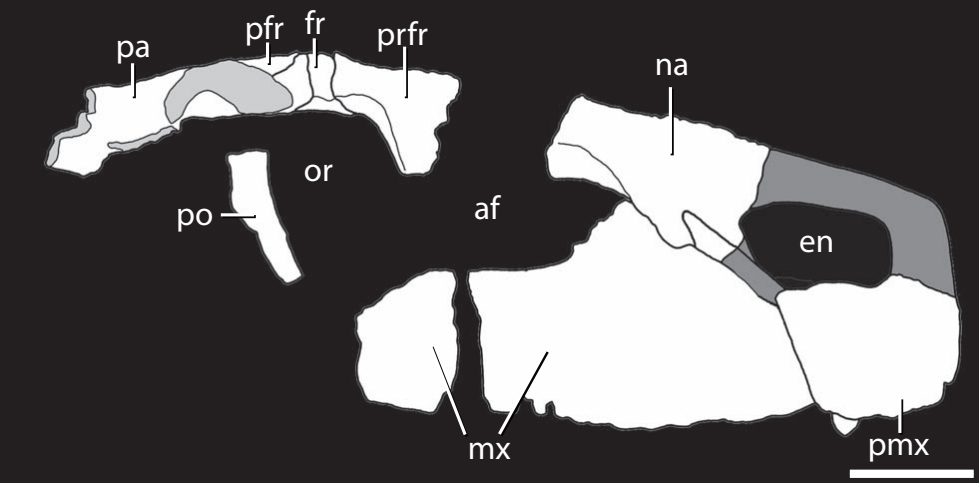
Reconstructed skull of the holotype of *Asperoris mnyama* (NHMUK PV R36615) in right lateral view. Light gray color indicates incomplete surfaces and dark gray colour hypothesizes the shape of the external naris based on the preserved potions of the nasal and premaxilla. Scale  = 5 cm. **Abbreviations:** af, antorbital fenestra; en, external naris; fr, frontal; mx, maxilla; na, nasal; or, orbit; pa, parietal; pfr, postfrontal; pmx, premaxilla; po, postorbital; prfr, prefrontal.

**Figure 3:**
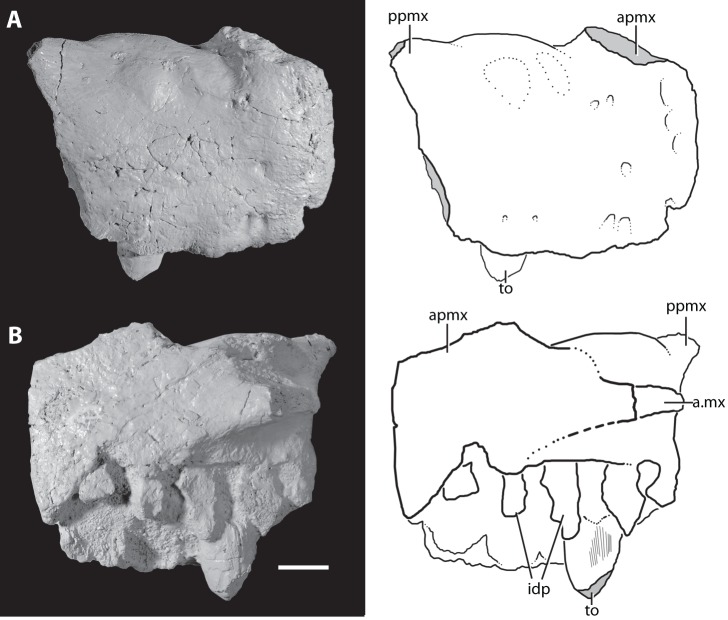
Right premaxilla of the holotype of *Asperoris mnyama* (NHMUK PV R36615). The premaxilla in lateral view (A) and interpretative drawing and medial view (B) and interpretative drawing. Light gray color indicates incomplete surfaces. Scale  = 1 cm. **Abbreviations:** a., articulates with; apmx, anterior process of the premaxilla; idp, interdental plates; mx, maxilla; ppmx, posterior process of the premaxilla; to, tooth.

**Figure 4:**
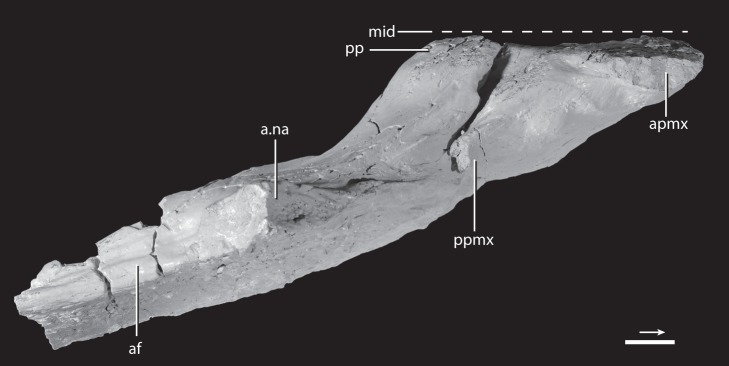
Premaxilla–maxilla articulation of the holotype of *Asperoris mnyama* (NHMUK PV R36615) in dorsal view. Scale  = 1 cm. Arrow indicates anterior direction. **Abbreviations:** a., articulates with; af, antorbital fenestra; apmx, anterior process of the premaxilla; mid, midline; na, nasal; pp, palatal process; ppmx, posterior process of the premaxilla.

**Figure 5:**
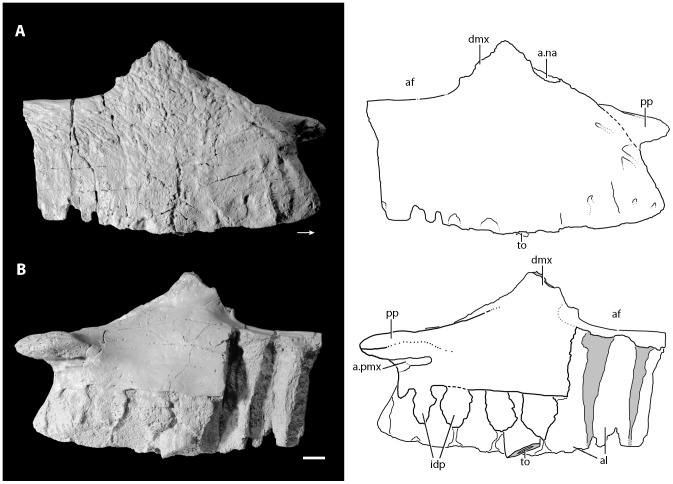
Anterior fragment of the right maxilla of the holotype of *Asperoris mnyama* (NHMUK PV R36615). The maxilla in lateral view (A) and interpretative drawing and medial view (B) and interpretative drawing. Light gray color indicates incomplete surfaces. Scale  = 1 cm. Arrow indicates anterior direction. **Abbreviations:** a., articulates with; af, antorbital fenestra; dmx, dorsal process of the maxilla; idp, interdental plates; na, nasal; pmx, premaxilla; pp, palatal process; to, tooth.

**Figure 6:**
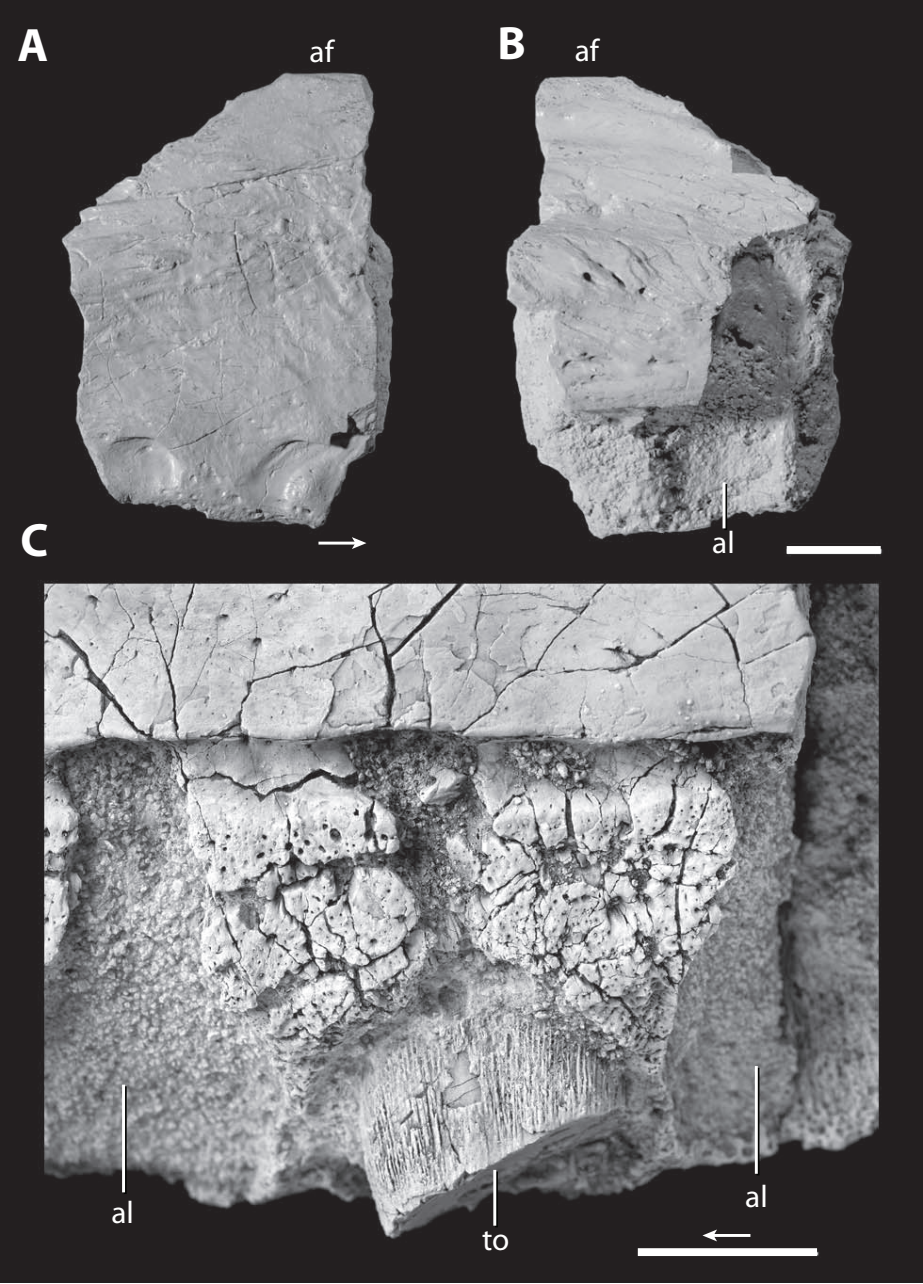
Posterior fragment of the right maxilla and close up of the base of broken maxillary tooth of the holotype of *Asperoris mnyama* (NHMUK PV R36615). Fragment in lateral (A) and medial (B) views and a medial (C) view of the base of a maxillary tooth. Scales  = 1 cm. Arrow indicates anterior direction. **Abbreviations**: af, antorbital fenestra; al, alveolus; to, tooth.

**Figure 7:**
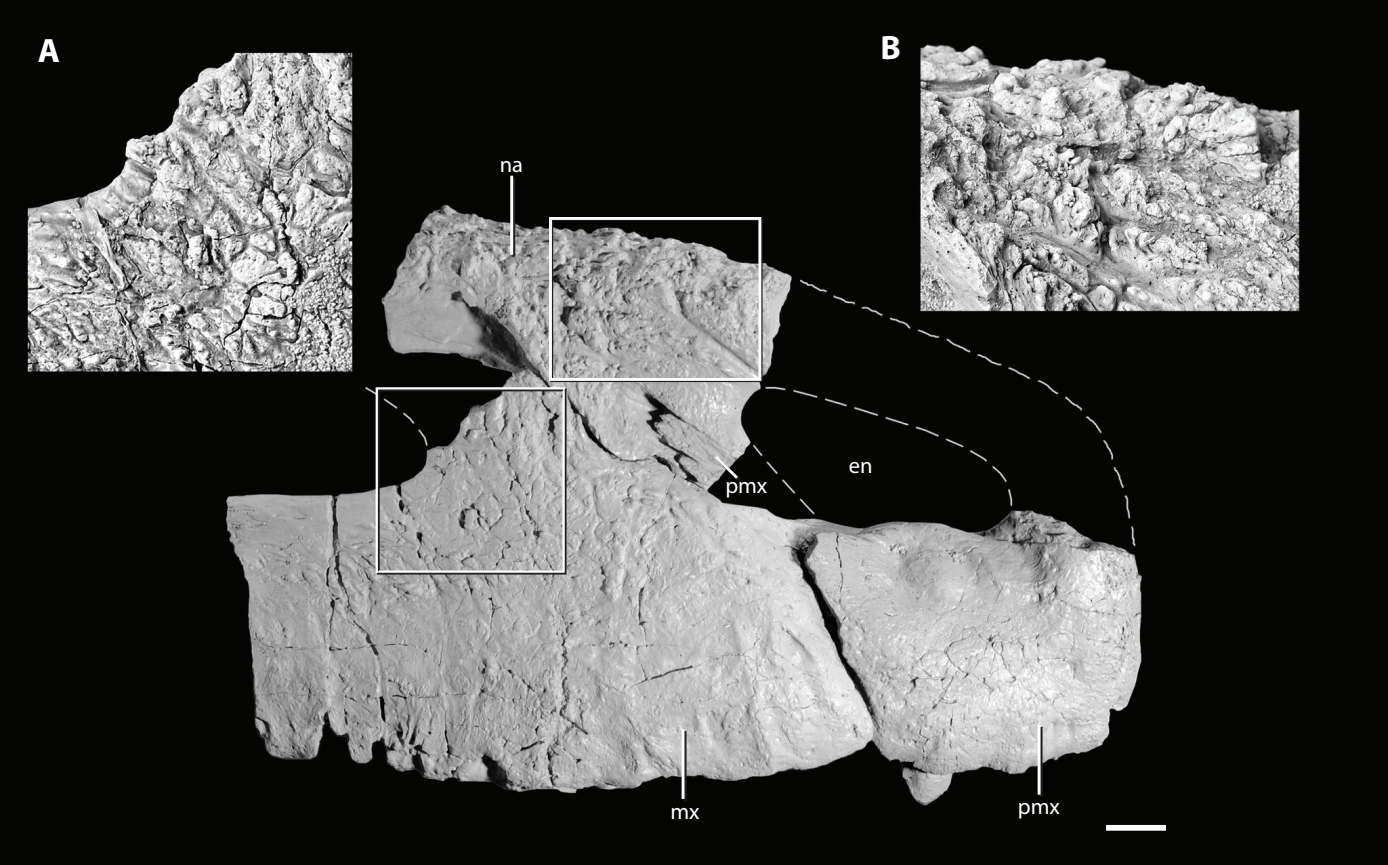
Unique sculpturing on the anterior portion of the skull of the holotype of *Asperoris mnyama* (NHMUK PV R36615) in right lateral view. Close up of the sculpturing on the dorsal process of the maxilla (A) and close up of the sculpturing of the nasal (B). Scale  = 1 cm. **Abbreviations**: en, external naris; mx, maxilla; na, nasal; pmx, premaxilla.

**Figure 8:**
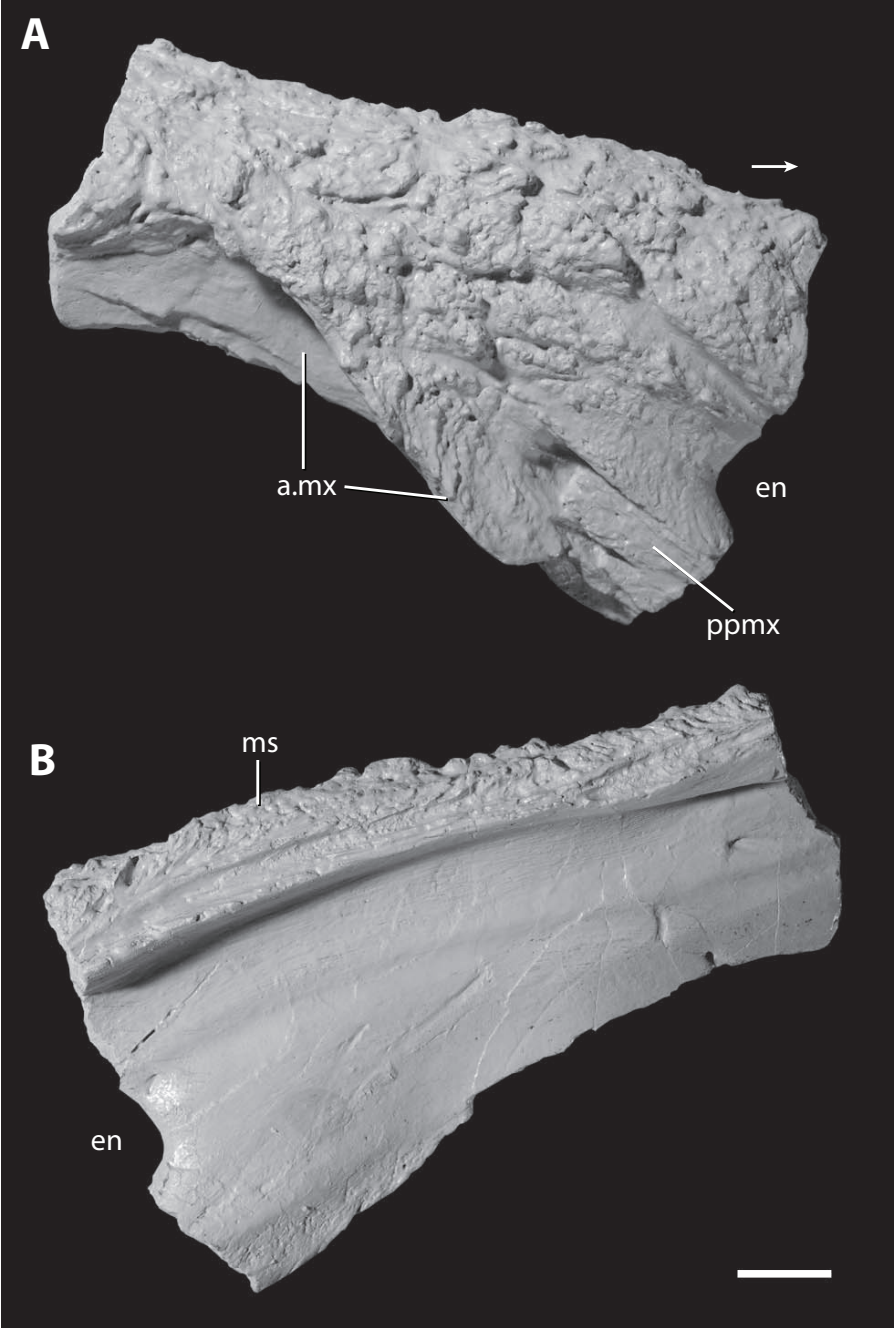
Right nasal of the holotype of *Asperoris mnyama* (NHMUK PV R36615). Nasal in lateral (A) and medial (B) views. Scale  = 1 cm. Arrow indicates anterior direction. **Abbreviations:** a., articulates with; en, external naris; ms, midline suture; mx, maxilla; ppmx, posterior process of the premaxilla.

**Figure 9:**
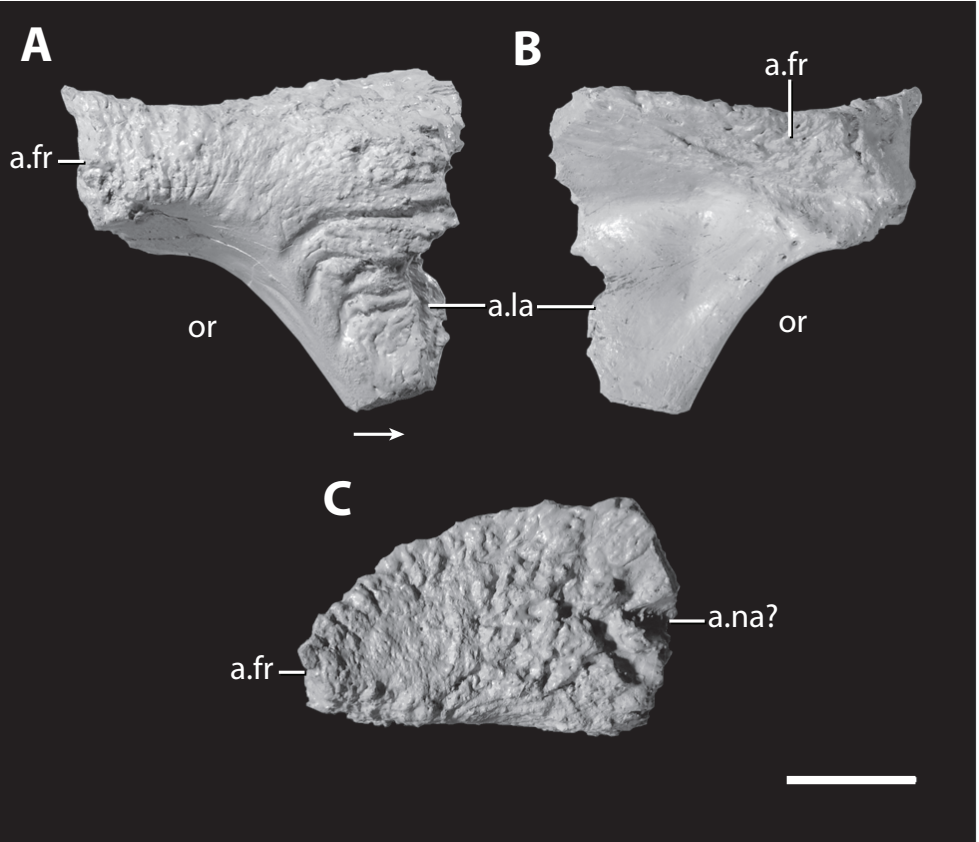
Right prefrontal of the holotype of *Asperoris mnyama* (NHMUK PV R36615). Prefrontal in lateral (A), medial (B), and dorsal (C) views. Scale  = 1 cm. Arrow indicates anterior direction. **Abbreviations:** a., articulates with; fr, frontal; la, lacrimal; na, nasal; or, orbit.

**Figure 10:**
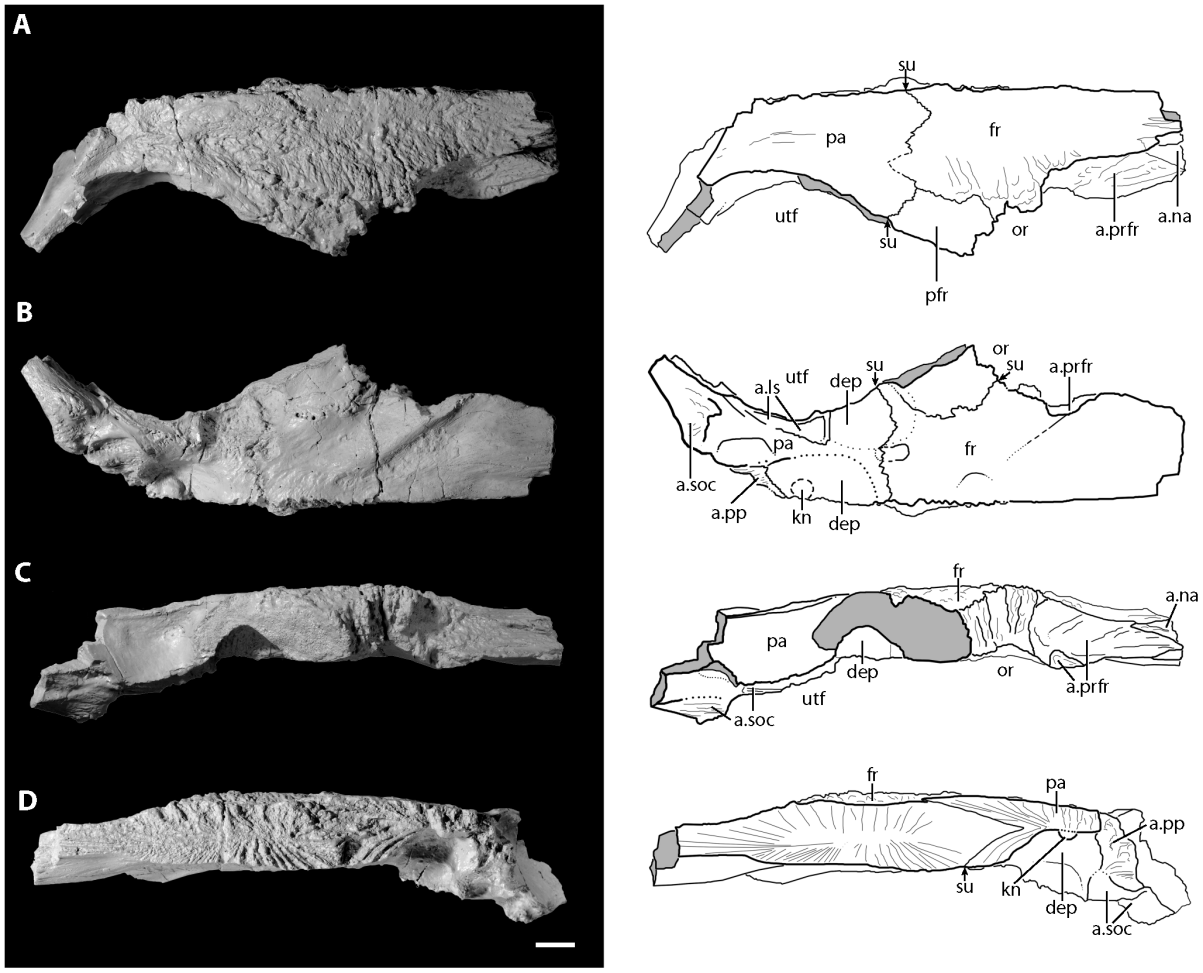
Right side of the skull roof of the holotype of *Asperoris mnyama* (NHMUK PV R36615) consisting of the frontal, postfrontal and parietal. Skull roof in dorsal view (A) and interpretative drawing, in ventral view (B) and interpretative drawing, in lateral view (C), and interpretative drawing, and medial view (D) and interpretative drawing. Light gray color indicates incomplete surfaces. Scale  = 1 cm. Arrow indicates anterior direction. **Abbreviations**: a., articulates with; dep, depression; fr, frontal; kn, knob; ls, laterosphenoid; na, nasal; or, orbit; pa, parietal; pfr, postfrontal; pp, postparietal; prfr, prefrontal; soc, supraoccipital; su, suture; utf, upper temporal fenestra.

**Figure 11:**
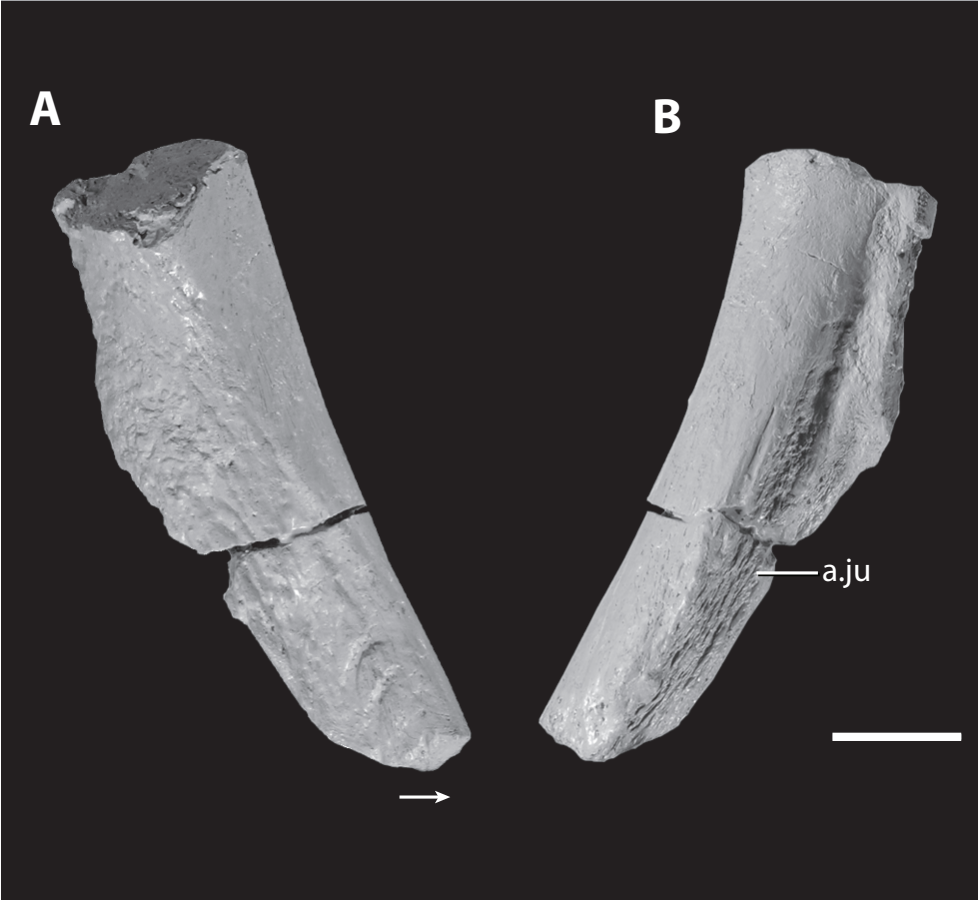
Incomplete postorbital of the holotype of *Asperoris mnyama* (NHMUK PV R36615). Ventral process of the postorbital in lateral (A) and medial (B) views. Scale  = 1 cm. Arrow indicates anterior direction. **Abbreviations:** a., articulates with; j, jugal.

**Figure 12:**
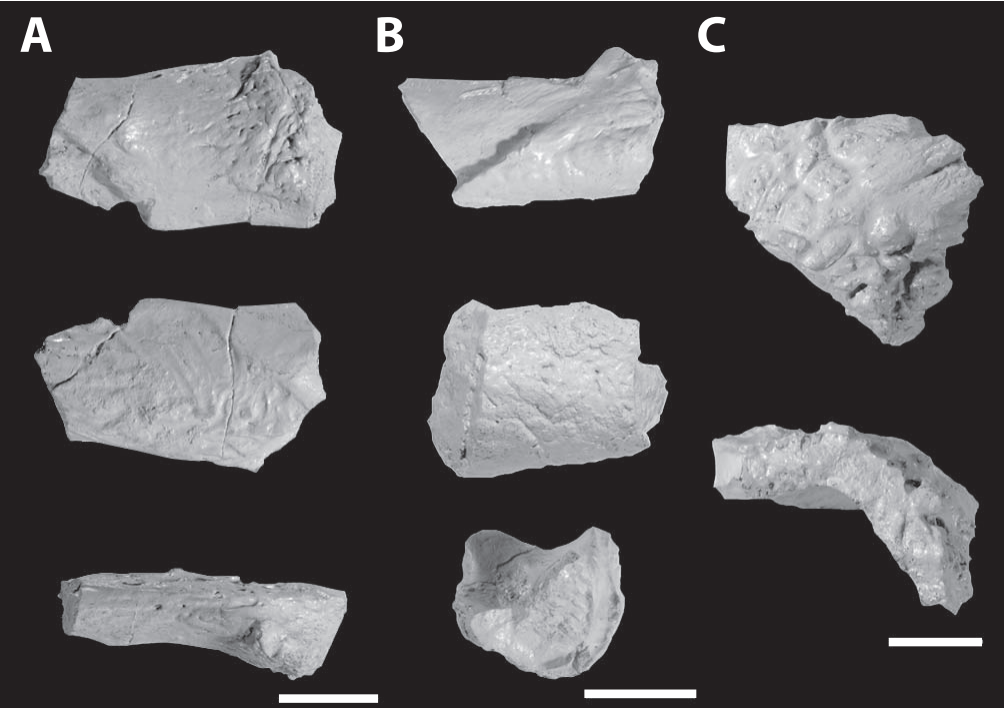
Unidentified skull elements of the holotype of *Asperoris mnyama* (NHMUK PV R36615). Skull fragments in three views in (A) and (B) and two views in (C). Scales  = 1 cm.

#### Stratigraphic horizon and locality

Lifua Member of the Manda beds, of early Middle Triassic (? late Anisian) age; locality U9/1, drainage of the Hita River between the Njalila and Hiasi rivers (exact locality not known) in the Ruhuhu basin, Songea district, southwestern Tanzania (Fig. 1). The locality (along with the taxon and associated taxonomic data) has been entered into the *Paleobiology Database* (www.paleodb.org) and is collection number 144368.

#### Diagnosis of the genus and species

*Asperoris mnyama* is a medium-sized (estimated skull length of single known specimen 50 cm) non-archosaurian archosauriform (see discussion). *Asperoris mnyama* has the following unique combination of cranial character states: posterodorsal process of the premaxilla fits into a distinct slot into the ventral process of the nasal (shared with *Erythrosuchus africanus* and other potential erythrosuchids); robust anteromedially directed palatal process of the maxilla (possible archosaur apomorphy); thecodont dentition (apomorphy of non-proterosuchid archosauriforms); the absence of an antorbital fossa on the maxilla anterior and ventral to the antorbital fenestra (plesiomorphy of Archosauriformes); dorsoventrally shallow antorbital fenestra; dorsoventrally thick skull roof; the absence of a parietal foramen or fossa (synapomorphy of the clade uniting *Vancleavea campi* + Archosauria within Archosauriformes in [8], [35]); and the possible presence of a postparietal element (plesiomorphy of Archosauriformes). *Asperoris mnyama* differs from all known archosauriforms in having highly sculptured cranial elements including the premaxilla, maxilla, nasal, prefrontal, frontal, postfrontal, and parietal, and in having a highly sculptured, dorsoventrally deep orbital margin of the frontal (Figs. 2–12).

In lacking an antorbital fossa at the base of the dorsal process of the maxilla and on the dorsal margin of the posterior process of the maxilla, the single known specimen of *A. mnyama* is demonstrably distinct from other archosauriforms with comparable elements from the Lifua Member of the Manda beds: “*Mandasuchus tanyauchen*” (NHMUK R6792), *Parringtonia gracilis* (NHMUK R8646), “*Pallisteria angustimentum*” (NHMUK R36620), and *Asilisaurus kongwe* (NMT RB159). *Asperoris mnyama* does not share directly comparable elements with *Stagonosuchus nyassicus* (GPIT/RE/3831), *Hypselorhachis mirabilis* (NHMUK R16586), an unpublished archosaur skeleton (NMT RB48), *‘Teleocrater rhadinus’* (NHMUK R6795), and *Nyasasaurus parringtoni* (NHMUK R6856), but the inferred phylogenetic position of *Asperoris mnyama* is not consistent with it belonging to any of these taxa.

### Description

#### Premaxilla

Most of the right premaxilla is preserved but it is missing the anterodorsal process and much of the posterodorsal process (Fig. 3). The lateral surface of the body is generally convex. There is a weakly developed narial fossa positioned ventral to the external naris, between the anterodorsal and posterior dorsal processes. This shallow depression is located at the anteroventral margin of the border of the naris and is demarcated by a weakly developed ridge. The narial fossa is much shallower than that described by Gower [36] for the premaxilla (SMNS 80260) of the loricatan archosaur *Batrachotomus kupferzellensis*. A groove that is present on the broken margin of the base of the anterodorsal process stretches posteriorly around the base to reach the margin of the premaxilla that borders the external naris. This groove terminates in a foramen that opens posterodorsally. Two rounded mounds positioned on the lateral surface just ventral to the border of the external naris likely represent the external expression of the most dorsal extent of the second and third tooth alveoli.

The lateral surface of the premaxilla is covered in a series of large and tiny foramina and a system of short ridges. The larger foramina are mostly restricted to the ventral margin and likely represent nutrient foramina. Two foramina are present on the lateral surface just ventral to the weak rim that defines the narial fossa. Additionally, there are four anteriorly opening foramina arranged in a vertical plane on the anterolateral surface, stretching from the base of the anterodorsal process to the ventral margin of the premaxilla. Tiny foramina occur on the anterior, ventral, and posterior parts of the lateral surface of the premaxilla with the highest concentrations nearest to the edges of the bone. The short ridges occur across the premaxilla, but are primarily concentrated at the anterior edge of the element and on the lateral surface of the posterodorsal process.

Only the ventral half of the posterior margin of the premaxilla is preserved but the articulation with the maxilla is clearly observed. The ventralmost portion of the posterior margin of the premaxilla is slightly laterally deflected. When the maxilla and premaxilla are in articulation, the anteroventral portion of the maxilla slightly overlaps laterally this ventral portion of the premaxilla. A similarly interlocking overlap does not appear to be common in archosauriforms, but is present in *Batrachotomus kupferzellensis* (SMNS 80260) in which it is more strongly developed. When placed in articulation, the premaxilla and the maxilla of *A. mnyama* fit relatively tightly together (Fig. 4), although there is a narrow, slit-like gap between the two elements. However, this gap between the premaxilla and the maxilla is substantially less well developed in *A. mnyama* than in *B. kupferzellensis* (SMNS 80260) and *Postosuchus kirkpatricki* (TTU-P 9000; [37], [38]) and there is no evidence on the posterior border of the premaxilla for a distinct border of a foramen (present in *B. kupferzellensis*). The dorsal half of the posterior margin of the premaxilla of *A. mnyama* is mediolaterally thinner than the ventral part, and partially laterally overlaps the maxilla.

The broken anterodorsal ( =  anterior dorsal) process is mediolaterally compressed and its anteroposterior length at its base is about half of the total length of the oral margin of the premaxilla. This process very likely separated the external nares along the midline. The mediolaterally compressed posterodorsal process of the premaxilla articulates with a distinct groove on the anterodorsal surface of the maxilla. The posterodorsal ( =  posterior dorsal) process projects posteriorly at about 45° to the horizontal in lateral view. The posterodorsal process is broken close to its base, and the main part of the body of the process is missing, but the tip of the process is preserved in a slot in the nasal. The tip of the posterodorsal process has a complex articulation with the nasal that has been revealed by slight disarticulation during preservation. The posterodorsal process of the premaxilla splits into three small fingers that have precise articulations with corresponding slots in the nasal. A tongue-in-groove articulation between the premaxilla and nasal is also present in *Erythrosuchus africanus* (BP/1/5207) and the archosaur *Revueltosaurus callenderi* [39].

Medially, much of the medial surface of the premaxilla is flat, marking the midline symphysis with its antimere (Figs. 3–4). Posteriorly, the medial surface is divided into a rimmed depression dorsally and a posteriorly directed palatal process. The depression represents a very closely matching articular surface for the palatal process of the maxilla. Ventral to this depression, the palatal process projects posteromedially, but is broken at its tip. This process, although broken, articulates with the maxilla ventral to the maxillary palatal process, and fits into a distinct slot in this element (see below). The interdental plates of the premaxilla are separated from the medial surface of the rest of the premaxilla by a distinct step that fades out posteriorly.

The premaxilla bears four alveoli separated from adjacent alveoli by triangular interdental plates. A rounded foramen is present between the dorsal margins of adjacent interdental plates, and the first three of these foramina are open ventrally. The alveoli are approximately equal in size.

#### Maxilla

The right maxilla is well-preserved in two sections that do not connect (Figs. 5–6). The larger of the two sections includes much of the anterior half of the maxilla with a partial dorsal ( =  ascending) process and complete palatal process, and the smaller section is from the posterior portion of the maxilla and preserves one complete alveolus and two partial alveoli. The rather flat lateral surface of the maxilla bears sculpturing similar to that of the frontal and nasal; however, this sculpturing is not evenly distributed across the surface of the maxilla. The most distinctive sculpturing is positioned just ventral and anterior to the antorbital fenestra (Fig. 7). Here, deep grooves radiate from the antorbital fenestra and surround small, rounded nubs of bone that extend further laterally beyond the level of the majority of the lateral surface of the maxilla. The ventral half of the lateral surface of the maxilla has less distinct sculpturing. Here the bone surface is uneven and consists of poorly differentiated knobs and seemingly irregularly oriented grooves. Tiny foramina also cover this area more abundantly near the ventral margin of the maxilla. This particular surface sculpturing is unique to *Aperoris mnyama*, but does have general similarities to the highly sculptured maxilla of the possible erythrosuchid *Guchengosuchus shiguaiensis* (IVPP V8808). A series of ventrally opening nutrient foramina lie close to the slightly convex ventral edge of the maxilla. Two similarly sized foramina are located 3 cm above the ventral edge of the maxilla near the contact with the premaxilla (Fig. 5). These large foramina open anteroventrally into anteroventrally trending grooves.

The surface of the maxilla for contact with the premaxilla slopes anteroventrally and is slightly anterodorsally convex in lateral view. The anterolateral termination of the maxilla tapers to a small tab that slightly overlaps the premaxilla laterally when in articulation (see above). In lateral view, the palatal process of the maxilla (hidden by the premaxilla when the two are in articulation) extends further anteriorly than the body of the maxilla. A clear facet for articulation with the posterodorsal process of premaxilla separates the palatal process from the rest of the maxilla; thus, the maxilla is excluded from the external naris by the posterodorsal process of the premaxilla. The anterodorsal surface of the dorsal process of the maxilla bears a clear, mediolaterally deep slot for articulation with the nasal. This slot is exposed in lateral view at its ventral termination. The architecture of the articulation between the nasal and the maxilla is precise; the grooves in the articular facet of the maxilla correspond directly to ridges present on the nasal (Fig. 7). Additionally, the articulation of the maxilla with the nasal continues anterior to the slot on the anterodorsal edge of the maxilla. Here a small splint of the nasal lies between the posterodorsal process of the premaxilla and the main body of the maxilla (Fig. 7–8).

The dorsal process of the maxilla is broken at its base; however, a few details are discernable. The process is mediolaterally broad anteriorly and tapers posteriorly toward the margin of the antorbital fenestra. The articulation of the nasal with the maxilla reveals that in lateral view the dorsal process of the maxilla projects at an angle of 45° to the horizontal at its base, but also that the process must be dorsoventrally short. This configuration further indicates that the anterior portion of the antorbital fenestra is not dorsoventrally deep. The posterior part of the base of the dorsal process does not have an antorbital fossa and this is consistent with the absence of an antorbital fossa along the ventral margin of the antorbital fenestra. The presence of an antorbital fenestra and the absence of an antorbital fossa alongside the ventral portion of the antorbital fenestra in *A*. *mnyama* is similar to that of non-archosaurian archosauriforms such as *Proterosuchus fergusi* (BP/1/3993), *Euparkeria capensis* (SAM-PK-5867), and *Guchengosuchus shiguaiensis* (IVPP V8808). However, the absence of any antorbital fossa at the base of the dorsal process clearly differentiates *A. mnyama* from *Erythrosuchus africanus* [11] and other possible erythrosuchids (e.g., *Chalishevia cotburnata*, PIN 4356/1; *Garjainia prima*, PIN 2394/5), and *Youngosuchus sinensis* (IVPP V3239; [40], [41]). The ventral margin of the antorbital fenestra is nearly horizontal in *A. mnyama*, suggesting that the body of the maxilla ventral to the antorbital fenestra maintained a broadly consistent dorsoventral depth along its length, similar to the condition in *Proterosuchus fergusi* (BP/1/3993), *Euparkeria capensis* (SAM-PK-5867), *Erythrosuchus africanus* (BP/1/5207), and some archosaurs such as *Postosuchus kirkpatricki* (TTU-P 9000). The height of the section of the posterior portion of the maxilla further supports the inference that the depth of the body of the maxilla ventral to the antorbital fenestra remains uniform along its length, and only tapered posteroventrally where the maxilla meets the jugal.

The convex medial surface of the maxilla is divided by a longitudinally oriented groove (referred to as the dental groove by [42]) trending subparallel to the tooth-bearing edge (Fig. 5). The surface of the body dorsal to the groove is smooth bone. Anteriorly, a horizontally oriented and robust palatal process extends anteriorly beyond the rest of the body of the maxilla, as in *Erythrosuchus africanus* (SAM-PK-K1098; [11]) and most archosaurs (e.g., *Postosuchus kirkpatricki*, TTU-P 9000). The palatal process of *Asperoris mnyama* tapers to a point anteriorly and it fits precisely into a broad and rimmed depression on the medial side of the premaxilla (see above). Dorsally, a posteriorly extending groove originates on the process, and defines the dorsal margin of the maxilla in medial view. Ventral to the palatal process, there is a clear facet on the medial surface of the maxilla for articulation with the posteriorly projecting palatal process of the premaxilla. In dorsal view, the palatal process of the maxilla arcs anteromedially and converges upon the midline. When placed in articulation with the premaxilla, it seems likely that the palatal process of the maxilla would have met its antimere at the midline (Fig. 4), given that the medial edge of the palatal process of the maxilla reaches a point level with the medial extent of the premaxilla. However, the medial surface of the maxillary palatal process lacks distinct facets or grooves for contact with its antimere. The posteroventral surface of the posterior portion of the palatal process may form the articular surface for the vomer. The ventral edge of the antorbital fenestra expands medially into a shelf and a shallow, longitudinally orientated groove lies on the dorsal margin of this shelf.

Unfused interdental plates separate the alveoli from the longitudinally oriented groove that bisects the medial surface of the maxilla. Incompletely delimited foramina are positioned ventral to and contacting the longitudinally oriented groove, and open ventrally at the anteroposterior midpoint of each alveolus. The lateral wall of each alveolus extends further ventrally than does the medial wall. As a consequence of this alveolar configuration, more of the apicobasal height of each tooth is exposed in medial view than in lateral view. The broken medial surfaces of the fifth and sixth alveoli indicate that the alveoli are deep, as further evidenced by the presence of only 3 to 4 mm of bone separating the antorbital fenestra from the dorsal end of the tooth sockets.

There is a minimum of 10 alveoli in the maxilla, of which seven are in the anterior section (Fig. 5) and three in the posterior section (Fig. 6). The first alveolus is the smallest and the diameter of the alveoli increase posteriorly. The alveoli decrease in diameter only at the very posterior preserved extent of the maxilla.

#### Nasal

The incomplete right nasal preserves the articular surface for the maxilla, the articular surface for the posterodorsal process of the premaxilla with the tip of the posterodorsal process preserved in articulation, and the posterior edge of the external naris (Fig. 8). The lateral and dorsal surfaces bear a complex sculpturing of knobs and grooves that is much more prominently developed than that on the maxilla. Within the complex sculpturing, a groove is present that originates at the posterior margin of the external naris and bifurcates posteriorly, anterior to the articular surface of the nasal with the maxilla (Fig. 8). Sculpturing is absent only immediately adjacent to the posterior margin of the external naris. A similar rugose sculpturing is also present on the nasal of *Guchengosuchus shiguaiensis* (IVPP V8808), but appears to be rare among most known non-archosaurian archosauriforms.

The preserved portion of the nasal has two anterior processes: an anterior process that would articulate with the anterodorsal process of the premaxilla, and a more ventral ( =  descending) process that extends posterior and ventral to the external naris. The more anterior process is broken at its base, slightly dorsal to the external naris, but appears to have extended anteroventrally. The more ventral process of the nasal bears a laterally open deep groove, within which fits the tip of the posterodorsal process of the premaxilla. As preserved, the posterior process of the premaxilla has been displaced slightly anteriorly from its original articulation with the nasal, revealing the complex contact between the two elements. Within the articular groove on the ventral process of the nasal, three slots are present that correspond to three short prongs on the tip of the posterodorsal process of the premaxilla. The cross section of the broken surface of the ventral process of the nasal, in anterior view, shows that a splint of the nasal separates the posterior edge of the tip of the posterodorsal process of the premaxilla from the maxilla, and that the nasal forms the complete medial wall of the articular surface for the posterodorsal process of the premaxilla. The insertion of the posterodorsal process of the premaxilla into the nasal is rare among archosauriforms, but does occur in the non-archosaurian archosauriforms *Erythrosuchus africanus* (BP/1/5207; [11]), *Guchengosuchus shiguaiensis* (IVPP V8808), *Chalishevia cotburnata* (PIN 4356/1), *Garjainia prima* (PIN 2394/5) and *Shansisuchus shansisuchus* (IVPP V2501; [43]), as well as in at least two archosaurs (*Turfanosuchus dabanensis*, IVPP V3237; *Revueltosaurus callenderi*; [39]). The relative size of the posterior processes of the premaxilla that fit into the nasal is much larger in *E. africanus* (BP/1/5207; [11]), *G. shiguaiensis* (IVPP V8808), and *C. cotburnata* (PIN 4356/1) than in *A. mnyama*.

In *Asperoris mnyama*, the outline of the external naris is inferred to have been anteroposteriorly elongated relative to its dorsoventral height, on the basis of the portions of the rim preserved on the nasal and premaxilla.

The ventral edge of the nasal preserves a complicated surface for articulation with the dorsal process of the maxilla. The most anterior portion of the nasal, posteroventral to the posterolateral process of the premaxilla, extends medial to and is partially hidden in lateral view by the maxilla when the two are in articulation. More posteriorly, a flange of the nasal anterolaterally overlaps the dorsal process of the maxilla, articulating with a groove on the dorsal process. Posterior to this, the dorsal process of the maxilla laterally overlaps the nasal, but the posterior extent of the articulation is not well defined and it is not clear if some of the nasal forms part of the antorbital fossa. This articular surface of the nasal is smooth and tapers ventrally.

Medially, the nasal bears a distinct midline sutural surface for its antimere that is about 1 cm deep. On the midline, a longitudinally oriented groove trends parallel to two thin ridges for the length of the preserved portion of the element. The midline surface also has several smaller grooves and ridges. Ventral to the midline sutural surface, the medial surface of the nasal is nearly smooth. A shallow groove originating at the posterior end of the external naris trends parallel to the midline suture for the entire preserved length of the nasal.

#### Prefrontal

The right prefrontal is nearly complete, missing only the tip of the ventral process (Fig. 9). The dorsal surface of the prefrontal has sculpturing that is similar in form and development to the dorsal surface of the nasal. The posterior portion of the prefrontal forms the anterodorsal part of the margin of the orbit. In this region, the sculpturing is composed of deep vertical grooves that are similar to those seen along the portions of orbital rim formed by the frontal and the postfrontal (see below). The prefrontal has an interdigitating suture posteriorly with the part of the frontal bordering the orbit. The prefrontal thins in dorsoventral depth anteriorly and dorsally overlaps the dorsal surface of the anterior portion of the frontal. A clear facet with short ridges and grooves on the ventral surface of the prefrontal represents the articular surface for the frontal.

The posteroventral portion of the prefrontal forms the anterior part of the internal border of the orbit. Here the bone surface is smooth and this surface is continuous with the fossa that borders the orbit on the ventral surface of the frontal. The lateral surface of the ventral process of the prefrontal is rugose with a few distinctive, nearly horizontally orientated grooves. The anterior surface of the ventral process forms a complex articular surface for the lacrimal. A dorsoventrally oriented groove, located on the anterior edge, likely matched a tongue of the lacrimal (not preserved). More dorsally, an anteriorly projecting tongue of the prefrontal laterally overlaps the lacrimal. The medial side of the ventral process is smooth.

#### Frontal

The complete right frontal is preserved in articulation with the postfrontal and the parietal (Fig. 10). The entire dorsal surface of the frontal bears fine, evenly distributed grooves, ridges, and foramina. A shallow, longitudinally oriented groove crosses the entire dorsal surface of the body of the frontal and continues onto the anterior half of the parietal. As a result, the midline area and the orbital margin are dorsally raised above the level of the middle of the frontal; the middle area is slightly depressed. The main body of the frontal is dorsoventrally deep (∼1.5 cm) and thins anteriorly.

Anteriorly, the frontal bears two articular surfaces, one for the prefrontal and one for the nasal. The prefrontal articular surface is laterally deflected, nearly flat, and bears a few low anteroposteriorly oriented ridges. The articular surface of the frontal for the nasal is located at the anterior margin of the element. The anteriormost portion of the frontal is missing, but it is clear that a finger of the nasal fitted into the frontal at the point where the dorsal surface of the frontal intersects the articular facet for the prefrontal. More medially, a finger of the frontal fitted into a groove on the dorsal surface of the nasal. There does not appear to have been long posteriorly directed processes of the nasals that extended between the frontals at the midline, unlike the condition in *Erythrosuchus africanus* (NHMUK R3592).

The frontal forms only a small section of the orbit margin in lateral view. The lateral surface, like that of the postfrontal and the prefrontal, is rugose and has dorsoventrally oriented ridges and grooves. A similar sculpturing of the orbit margin is present in *Batrachotomus kupferzellensis* (SMNS 80260) and Nesbitt et al. [44] argued that a palpebral element may have articulated with this surface. However, in *Asperoris mnyama* there is not a clear facet for articulation with any other element.

Posteriorly, the frontal forms interdigitating sutures with the postfrontal laterally and the parietal posteriorly. In dorsal view, a finger of the frontal articulates into the parietal laterally, and more medially a finger of the parietal enters the body of the frontal (Fig 10A). In medial view, a posterior process of the frontal extends into the body of the parietal, such that the parietal both under- and overlies the posterior portion of the frontal (Fig 10D). The entire midline edge of the frontal forms a sutural surface for its antimere. Here, grooves and ridges radiate from the center of the element (Fig 10D).

Ventrally, most of the surface of the frontal is smooth. A rounded ridge marks the anteromedial edge of the fossa bordering the orbit, and a shallow groove parallels the orbit fossa rim on the anteromedial side. The anterior portion of the frontal is ventrally bowed transversely. The posterior portion of the ventral surface forms an almost transverse, interdigitating suture with the parietal. A small splint of the frontal extends between the postfrontal and parietal (Fig. 10B). A deep pit that is defined by a clear rim is located on the suture between the parietal and the frontal, and the frontal contributes to the large depression formed by the postfrontal, frontal, and parietal.

#### Postfrontal

Much of the right postfrontal is present with the exception of the articular surface for the postorbital (Fig. 10). The element is dorsoventrally thick, and has a similar depth and a similar pattern of vertically oriented grooves and ridges to the orbit margin of the frontal. The medial surface of the postfrontal forms interdigitating sutures with both the parietal and the frontal. Dorsally, the surface of the postfrontal is strongly rugose, similar to that of the frontal and nasal. In ventral view, the surface of the prefrontal is mostly smooth and the posterior portion of the element contributes to the anterior border of a deep depression that continues onto the parietal and frontal. The interdigitating suture between the frontal and postfrontal is distinctly raised relative to the surrounding bone.

#### Parietal

Most of the right parietal is preserved except for the posterolateral end of the squamosal (occipital) process (Fig. 10). Anteriorly, the parietal has a complex articulation with the frontal (see above) and the lateral edge of the element forms the medial portion of the upper temporal fenestra. It is not clear if and how the parietal contacted the postorbital.

The rugose dorsal sculpturing on the frontal continues onto the parietal. The dorsal surface of the parietal is essentially flat and there is no parietal foramen on the midline or any indication of the pineal fossa seen in, for example, *Erythrosuchus africanus* [11]. Additionally, the lateral and posterior edges of the dorsal surface of the parietal slightly overhang the upper temporal fenestra and the posterior surface of the parietal, respectively. The posterior edge of dorsal surface of the parietal is raised relative to the surface of the main body, as in *Youngosuchus sinensis* (IVPP V3239). In lateral view, the medial wall of the upper temporal fenestra is dorsoventrally deep (20 mm) and distinctly concave in lateral view.

Posteriorly, the parietal slopes posteroventrally to form the occipital surface. A broken parietal tubercle is present near the midline on the occipital surface, as in *Erythrosuchus africanus* [11]. An interdigitating sutural surface just lateral to the midline suture of the parietal suggests that a postparietal might have been present but was not preserved with the specimen. A pocket that marks the articulation of the parietal with the dorsal surface of the supraoccipital lies ventral to the possible articular surface for the postparietal. The squamosal/occipital process of the parietal expands posterolaterally, but the surface for articulation with the squamosal is not preserved. Ventrally, this process would have articulated with the dorsal edge of the paroccipital process. It is not clear if a posttemporal opening was present.

Ventrally, the parietal bears three distinct fossae, one that also extends onto the frontal and postfrontal and which is positioned near the lateral edges of all three elements, one positioned on the interdigitating suture between the frontal and the parietal, and one positioned entirely within the parietal and bisected by the midline suture. This last fossa contained a well-developed knob of bone at its center. An anteroposteriorly elongated facet on the lateral edge of the ventral surface of the parietal possibly represents an area of contact with the laterosphenoid.

#### Postorbital

Only the ventral process of the right? postorbital is preserved (Fig. 11). The element tapers ventrally and bears a depression with a rugose sculpturing on the anterolateral side of the ventral end. A facet for the dorsal process of the jugal is present on the posterovental side of the element, and this facet is divided into two shallow and parallel basins. The more lateral basin is visible in posterolateral view. The orbital margin is marked by small grooves. Proximally, the cross section of the element is triangular whereas more ventrally the cross section becomes more oval.

#### Other skull fragments

Three skull fragments were collected along with the holotype but cannot be identified (Fig. 12A–C). All three likely belong to the skull given that they share a similar preservation style and similarities of surface sculpturing with the other skull elements. One fragment (Fig. 12A) might be the pterygoid process of the basisphenoid, whereas the other two (Fig. 12B–C) likely represent dermal elements given that both have the distinct kind of sculpturing that is also present on the skull table, premaxilla and maxilla.

#### Dentition

Only two broken teeth are preserved in the holotype of *Asperoris mnyama*: a single premaxillary tooth in the third alveolus (Fig. 3), and a single maxillary tooth in the fourth alveolus (Fig. 4). Both teeth are fully erupted, with the base of the crown as well as the part of the root preserved. The preserved enamel of the premaxillary tooth is smooth, but there is not enough of the crown preserved to determine if serrations were present. The base of the premaxillary tooth is circular in cross section whereas that of the maxillary tooth is oval, with the anteroposterior axis longer than the mediolateral axis. The dentition is seemingly thecodont given that each tooth sits in a deep socket and is not directly attached to the surrounding bone. Furthermore, there is no indication that the surrounding bone is heavily remodelled as in the ankylothecondont non-archosaurian archosauriforms *Proterosuchus fergusi* (BP/1/3993) and *Guchengosuchus shiguaiensis* (IVPP V8808). However, the base of each tooth of *A. mnyama* bears dorsoventrally oriented grooves and ridges that are reminiscent of similar grooves and ridges present in these ankylothecondont archosauriforms. In these taxa, the grooves and ridges attach the base of the tooth directly to the premaxilla, maxilla, or dentary whereas the grooves and ridges at the base of the teeth of *A. mnyama* clearly do not attach to the premaxilla or maxilla.

### Phylogenetic analysis

**Figure 13:**
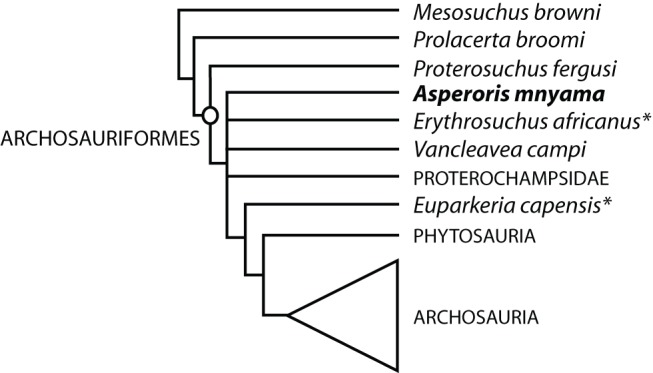
The relationships of *Asperoris mnyama* (NHMUK PV R36615) among archosauromorphs. Strict consensus tree of 720 MPTs (TL = 1288, CI = 0.374, RI = 0.775). Asterisks indicate the alternative sister-taxon relationships of *Asperoris mnyama* in the source MPTs. Species level taxa were collapsed into larger clades (clade name in capitals) and the interrelationships within these clades is identical to that presented by Nesbitt (2011).

Our analysis found 720 most parsimonious trees (MPTs) of length 1288 (consistency index [CI]  = 0.374, retention index  = 0.775). In the strict consensus tree (Fig. 13), *Asperoris mnyama* was recovered as a non-archosaurian archosauriform in a polytomy with *Erythrosuchus africanus*, *Vancleavea campi*, Proterochampsidae, *Euparkeria capensis* and Phytosauria + Archosauria. In all of the MPTs, *A. mnyama* is found as either the sister taxon of *Erythrosuchus africanus* or of *Euparkeria capensis*. The relationship between *A. mnyama* and *Erythrosuchus africanus* is supported by the presence of an articulation of the posterodorsal process of the premaxilla into the nasal (character 4, state 3) whereas the relationship between *A. mnyama* and *Euparkeria capensis* is supported only by archosauriform plesiomorphies (character 146, state 0) or highly homoplastic character states (character 27, state 1; CI = 0.154). Inclusion of *A. mnyama* within Archosauria occurs only in trees at least four steps longer than MPTs.

## Discussion

Our phylogenetic analysis resolves *Asperoris mnyama* as a non-archosaurian archosauriform. The presence of an antorbital fenestra and the absence of a parietal foramen places *A. mnyama* within archosauriforms given that these character states have been repeatedly found as synapomorphies of Archosauriformes or less inclusive clades in nearly all phylogenetic analyses of archosauromorphs [4], [8], [35], [45]–[49]. Fully thecodont dentition furthermore suggests that *A. mnyama* lies within or as the sister taxon to the least inclusive clade including *Erythrosuchus africanus* and Archosauria. The preserved anatomy of *A. mnyama* appears to preclude an assignment to Archosauria based on the absence of an antorbital fossa on the main body of the maxilla and the likely presence of a postparietal at the posterior portion of the skull table.

The lack of preservation of much of the skull and all of the mandibular and postcranial skeleton of *A. mnyama* and the absence of most stem archosaurs in our phylogenetic dataset precludes a precise phylogenetic resolution of the new taxon among non-archosaurian archosauriforms. However, our analysis did recover a sister taxon relationship between *A. mnyama* and *Erythrosuchus africanus* in some of the most parsimonious trees, and this relationship is supported by the presence of a posterodorsal process of the premaxilla that inserts into the ventral process of the nasal. The presence of this character state is restricted to *A. mnyama* and *E. africanus* among early archosauriforms in this analysis but has a wider distribution among other early archosauriforms not included in our phylogenetic analysis. For example, the posterodorsal process of the premaxilla inserts into the ventral process of the nasal in *Guchengosuchus shiguaiensis* (IVPP V8808), *Chalishevia cotburnata* (PIN 4356/1), *Garjainia prima* (PIN 2394/5) and *Shansisuchus shansisuchus* (IVPP V2501). Currently, it is not clear if all of these taxa are more closely related to *E. africanus* than to other archosauriforms (i.e., Erythrosuchidae, see [50] for support of monophyly) or to some extent form a grade, as recently suggested by Ezcurra et al. [51] (see [10]). If these taxa represent a grade, the presence of the posterodorsal process of the premaxilla that inserts into the ventral process of the nasal would not support *A. mnyama* as a particularly close relative of *E. africanus*.

Despite the nature of the premaxilla-nasal articulation, *Asperoris mnyama* has some character states that can be used to argue against an erythrosuchid affinity. For example, the parietals of *A. mnyama* are flat like those of, for example, *Vancleavea campi* (GR 138) and *Euparkeria capensis* (SAM-PK-5867) rather than having a depression on their dorsal surface of the parietals at the midline ( =  pineal fossa of [50]) such as is found in *Erythrosuchus africanus* [11], [50], *Guchengosuchus shiguaiensis* (IVPP V8808), *Garjainia prima* (PIN 2394/5), and *Shansisuchus shansisuchus* (IVPP V2504; [43]). *Youngosuchus sinensis* (IVPP V3239; [50]) lacks a pineal fossa, but its status as a possible erythrosuchid has been questioned [40]. *Asperoris mnyama* also lacks the small antorbital fossa at the posterior portion of the base of the dorsal process of the maxilla that is present in *E. africanus* [11], *Chalishevia cothurnata* (PIN 4356/1), *G. prima* (PIN 2394/5) and *Y. sinensis* (IVPP V3239). Thus, the case that *A. mnyama* is an erythrosuchid is far from compelling.

The enlarged palatal process of the maxilla of *Asperoris mnyama* perhaps suggests a phylogenetic position closer to Archosauria. Early archosauriforms such as *Proterosuchus fergusi* (NMQR 880) completely lack palatal processes, and taxa such as *Guchengosuchus shiguaiensis* (IVPP V8808) have a palatal process that is poorly differentiated from the body of the maxilla. *Erythrosuchus africanus* [11] and *Euparkeria capensis* (SAM-PK-5060) have a somewhat developed palatal process of the maxilla, but this process might not meet its antimere at the midline. In contrast, most members of Archosauria have large palatal process that meet their antimeres at the midline [8]. In these taxa (e.g. *Postosuchus kirkpatricki*, TTU-P 9000), the palatal process extends medially to reach the midline and, in disarticulated maxillae, the palatal processes have ridges and grooves that mark the midline articulation. The condition in *A. mnyama* is somewhat intermediate between that of non-archosaurian archosauriforms and archosaurs. The robust palatal process expands medially to near the midline, but it is not clear that it actually contacted its antimere and the medial surface of the palatal process does not have any indication of being a contact surface.

In sum, the available evidence suggests that *Asperoris mnyama* is either a non-archosaurian archosauriform that is closer to *Erythrosuchus africanus* than to other archosauriforms (i.e., possibly an erythrosuchid) or is slightly phylogenetically closer to Archosauria than is *E. africanus*. Regardless of the correct phylogenetic position, *A. mnyama* is the first confirmed non-archosaurian archosauriform among a diverse archosauriform fauna from the Lifua Member of the Manda beds.

Archosaurs and non-archosaurian archosauriforms overlapped temporally throughout the Early and Middle Triassic, as predicted by ghost lineages determined from recent phylogenies (e.g., [8], [15], [52]), suggesting that associations of these taxa should be commonplace. However, localities or a set of localities within a single sedimentary package (e.g., member or formation) where both stem archosaurs and archosaurs co-occur are relatively rare. Associations do occur in the Lower Triassic in Russia, close to the Lower to Middle Triassic boundary in China, and in the Middle Triassic of Russia, China, South America, and possibly in North America (see table 2). In the Late Triassic, proterochampsids and early dinosaurs occur in the same area (Ischigualasto Formation) in Argentina [53] and possibly in the Santa Maria sequence of southern Brazil, whereas in North America *Vancleavea campi* occurs with early dinosaurs throughout deposition within the Chinle basin [35], [54], [55]. Moreover, if phytosaurs fall outside of Archosauria (see [8]), then the co-occurrence of non-archosaurian archosauriforms (i.e., phytosaurs) and archosaurs is common in North America, Europe, northern Africa, and India throughout the Late Triassic and persisted until the end-Triassic mass extinction event.

**Table 2:** Non-archosaurian archosauriform (top) and archosaur (bottom) associations in the Early–Middle Triassic.

Temporal bin	Location	Taxon pairs
Lower Triassic		
	Russia (Yarenskian Gorizont)	
		*Garjainia prima* [56]
		*Vytshegdosuchus zheshartartensis* [12], [57]
Lower Triassic/Middle Triassic		
	China (lower Heshanggou Formation)	
		*Proterosuchus* [14]
		*Xilousuchus sapingensis* [14], [15]
Middle Triassic		
	China (upper Ermaying Formation)	
		*Shansisuchus shansisuchus* [43]
		*Wangisuchus tzeyii* [14], [43]
	South America (Chañares Formation)	
		*Chanaresuchus bonapartei* [58]
		*Gracilisuchus stipanicicorum* [59]
	South America (Santa Maria sequence 1)	
		*Archeopelta arborensis* [60]
		“rauisuchian” [60]
	North America (Moenkopi Formation)	
		non-archosaurian archosauriform [61]
		*Arizonasaurus babbitti* [62]–[64]
