# Correction: A New Archosauriform (Reptilia: Diapsida) from the Manda Beds (Middle Triassic) of Southwestern Tanzania

**DOI:** 10.1371/journal.pone.0093657

**Published:** 2014-03-24

**Authors:** The PLOS ONE Staff

**Notice of Republication**

This article was republished on March 7, 2014 to correct for formatting issues in Table 1. The publisher apologizes for these errors. Please download this article again for the correct version. The originally published, uncorrected article and the republished, corrected article are provided here for reference.

## Supporting Information

File S1 **Originally published, uncorrected article.**
(PDF)

File S2 ** Republished, corrected article.**
(PDF)

## References

[1] Nesbitt SJ, Butler RJ, Gower DJ (2013) A New Archosauriform (Reptilia: Diapsida) from the Manda Beds (Middle Triassic) of Southwestern Tanzania. PLoS ONE 8(9): e72753 doi:10.1371/journal.pone.0072753 24086264 10.1371/journal.pone.0072753 PMC3785487

